# Utility of Subjective Sleep Assessment Tools for Healthy Preschool Children: A Comparative Study Between Sleep Logs, Questionnaires, and Actigraphy

**DOI:** 10.2188/jea.JE20090054

**Published:** 2010-03-05

**Authors:** Mizue Iwasaki, Sachiko Iwata, Akiko Iemura, Natsumi Yamashita, Yasushi Tomino, Tokie Anme, Zentaro Yamagata, Osuke Iwata, Toyojiro Matsuishi

**Affiliations:** 1 Japan Children’s Study Group, Research Institute of Science and Technology for Society, Japan Science and Technology Agency, Tokyo, Japan; 2 Institute for Developmental and Cognitive Neuroscience, Department of Pediatrics and Child Health, Kurume University School of Medicine, Kurume, Fukuoka, Japan; 3 Centre for Perinatal Brain Research, Institute for Women’s Health, University College London, United Kingdom; 4 Centre for Bio-Statistics, Kurume University School of Medicine, Kurume, Fukuoka, Japan; 5 University of Tsukuba, Graduate School, Comprehensive Human Sciences, Tsukuba, Ibaraki, Japan; 6 Department of Health Sciences, School of Medicine, University of Yamanashi, Chuo, Yamanashi, Japan

**Keywords:** actigraphy, sleep log, questionnaire, preschool child, sleep pattern, subjective sleep assessment, parental report

## Abstract

**Background:**

Sleep pattern is an important factor in a child’s mental, behavioural and physical status. To evaluate the sleep patterns of children, subjective tools such as sleep logs and questionnaires are still widely used in addition to objective methods of sleep assessment. Despite the established correlation between subjective and objective sleep variables, the characteristic features of subjective assessment have not been elucidated.

**Methods:**

To investigate the characteristics of parental sleep assessment (daily sleep log and brief questionnaire) in preschool children, a 7-day actigraphic sleep study was conducted in 48 healthy 5-year-old children.

**Results:**

Sleep schedule variables in the parental reports generally correlated well with actigraphic assessment of sleep patterns; however, sleep periods were longer in parental reports than in actigraphic recordings. Although the daily sleep log was better correlated with actigraphy, the brief questionnaire showed a good correlation with sleep pattern on weekday actigraphic assessments. Parental reports recorded fewer than 10% of the night wakings recorded by actigraphy.

**Conclusions:**

Subjective sleep assessments remain useful, although their utility depends on the purpose and size of the study in question. However, knowledge of the potential biases and characteristics of such assessments is essential for correct interpretation of the data.

## INTRODUCTION

Sleep problems in childhood are usually noticed only after significant changes in a child’s behavior, mood, or performance.^1^^,^^2^ Even when parents notice a sleep problem with their child, fewer than half discuss the issue with their pediatrician.^3^ However, sleep problems and disorders in children are directly associated with their mental and physical status.^4^^–^^7^ Hence, a precise assessment of a child’s sleep patterns is important.

Although polysomnography has been the gold standard for evaluating sleep problems, its availability remains limited because it requires expensive equipment and considerable expertise. In addition, polysomnography requires the child to sleep in a laboratory, which might be burdensome for young children and hence more likely to alter their sleep patterns.^8^ Over the past 2 decades, actigraphy has been developed and standardized to provide an objective indirect estimation of sleep patterns from limb motions. Actigraphs are handy ankle/wrist devices that continuously record limb motion over a period of weeks within the child’s natural environment.^9^^–^^11^ The American Academy of Sleep Medicine has recently recommended the use of actigraphy as a sleep assessment tool for a wide range of people, including adolescents and children with or without pathological conditions.^12^ However, actigraphic studies that address the sleep patterns of preschool children are limited.^13^^–^^15^ Despite the increasing availability of these objective assessment tools, subjective assessment tools such as questionnaires and sleep logs are still widely used. Although simple questionnaires are suitable for screening and surveillance of a large population,^5^^,^^16^^,^^17^ sleep logs are preferred for more detailed assessment of sleep patterns, admittedly at the expense of increased burdens on participants and their parents.

Although it is a great advantage to have a wide range of sleep assessment tools, caution is required when comparing the results obtained by different methods. Several studies have reported significant linear correlations in sleep schedule variables between parental reports and actigraphy in young children. However, parental observations are believed to be less sensitive in assessing sleep quality variables (such as night wakings) than in assessing sleep schedule variables.^14^ The accuracy of parental reports depends on the quality and continuity of the observation, which vary according to the punctuality of the parents and the lifestyle of the family. Sadeh observed that the discrepancy between parental and actigraphic observations increased over a period of weeks, because parents grew tired or less motivated with time.^18^ A better understanding of the characteristics of each sleep evaluation tool is urgently required.

The aim of this study was to use actigraphy to investigate the characteristics and potential biases affecting parental sleep assessments of healthy 5-year-old children. We hypothesized that sleep assessments based on parental observation would identify longer sleep periods than those recorded on actigraphy.

## METHODS

This study was conducted under the guidance and approval of the Ethical Committee of Kurume University School of Medicine. Written informed consent was provided by a parent of each child participating in the study.

### Study population

In February 2006, an introductory letter regarding the study was sent to the parents of 48 five-year-old children (27 boys and 21 girls) who were randomly chosen from the registers of 2 private day-care nurseries in Kurume, Fukuoka, Japan. Ultimately, the parents of all 48 children agreed to participate in the study. One nursery (nursery A, *n* = 25) provided short-term care between 9:30 AM and 1:30 PM, from Monday through Friday, for families with in-home daytime caregivers (family or professional). The other (nursery B, *n* = 23) provided longer and more flexible care between 7:00 AM and 8:00 PM, from Monday through Saturday, for families without in-home daytime childcare. As part of their preparation for school life, all children, except for 1 girl in nursery B, had been weaned off daytime naps by the time of the study (the data from this girl were not analyzed further because of the known effect of naps on sleep patterns^19^). Ultimately, 47 children participated in a 7-day data collection from a Friday afternoon to the next Friday afternoon.

### Actigraphic measurement

During the study period, children were asked to wear an actigraph watch (Ambulatory Monitoring, Ardsley, NY, U.S.A.) on their wrist (either dominant or non-dominant side) except during bathing. The children’s activity was recorded using the zero-crossing mode in 1-minute epochs, and the acquired data were analysed using Motion Logger ActFAST Analysis Software (Ambulatory Monitoring). Bedtime, sleep onset time, number of night wakings (>5 minutes), and sleep end time were defined according to published algorithms.^20^ In addition, sleep latency was defined as the period between bedtime and sleep onset time, whereas sleep period was defined as that between sleep onset time and sleep end time (Online supplementary 1). The data were manually inspected for invalid records by 2 experienced investigators (M.I. and Y.T); data were excluded by consensus.

### Sleep log

Parents were requested to record a daily sleep log and complete a brief questionnaire at the end of the study period. Sleep/wake status was recorded in the daily sleep log, including sleep onset time, sleep end time, sleep period, and number of night wakings (>5 minutes) (Online supplementary 1). For periods when children were staying at the nursery, the sleep log was recorded based on the information provided by their caretakers.

### Questionnaire

The brief questionnaire consisted of 12 major questions regarding the child’s sleep patterns, sleep quality, and environmental background, which were based on a brief infant sleep questionnaire^17^ (Online supplementary 2). For sleep variables, the questionnaire asked about the usual bedtime, sleep onset time, sleep end time, sleep period, and number of night wakings (>5 minutes) based on the wake-sleep cycle over the past 4 weeks, without distinguishing between weekdays and weekends. Sleep latency was also calculated from bedtime and sleep onset time.

### Statistical analysis

The values obtained for bedtime, sleep onset time, sleep latency, sleep end time, and number of night wakings from the 3 assessment tools were averaged for weekdays, the weekend, and the entire study period for each individual (bedtime and sleep latency data were not collected in the sleep log). For children not attending the nursery on Saturday (ie, all children from nursery A and 8 children from nursery B), weekdays were defined as the period from Sunday night to Friday morning (weekends were from Friday night to Sunday morning). For children attending nursery on Saturday (14 children from nursery B), weekdays were defined as the period from Sunday night to Saturday morning (weekends from Saturday night to Sunday morning). Sleep variables from actigraphy and sleep logs were averaged for weekdays, weekends, and the whole of the study period, and these data were compared with each other and with the questionnaire (variables from the questionnaire for the whole study period were compared with data from other assessment tools over the 3 study periods; see the Questionnaire section in the Methods for more detail) using analysis of variance and Pearson’s correlation coefficient.

Because of the exploratory nature of this study, p-values for comparisons over multiple study parameters are shown without correction, and a p-value less than 0.008 was considered to indicate statistical significance. Bonferroni correction was used to adjust for multiple comparisons for the 6 sleep variables, but not for other parameters.^21^^,^^22^

## RESULTS

Actigraphy was ultimately tolerated by all participants. All invalid actigraphic data were confirmed—on the basis of entries in sleep logs—to be recordings taken during bathing or swimming, when children were not wearing actigraphs. Six children were sometimes absent from the nursery because of illness; the mean number of days absent (standard deviation) was 2.7 (1.6). The dates they were absent were excluded from the analysis.

### Comparison of the questionnaire and sleep log

The sleep onset time, sleep end time, and sleep period in the sleep logs were linearly correlated with the respective values on the questionnaire for weekdays and for the whole study period (*P* < 0.001 for all comparisons). As for weekends, a significant correlation was observed only for sleep end time (*P* = 0.003); no correlation was observed in the number of night wakings, regardless of the study period (Table 1). There was no significant difference in sleep variables between the questionnaire and the sleep log (Table 2), except that the number of night wakings was higher on the questionnaire than on the sleep log, both for the whole study period and for weekdays (*P* = 0.006 and *P* = 0.003, respectively).

**Table 1.** Correlations in sleep variables between sleep logs, questionnaires, and actigraphy

	Sleep log vs. Questionnaire			Actigraphy vs. Sleep log			Actigraphy vs. Questionnaire		
			
	Overall	Weekday^a^	Weekend^a^	Overall	Weekday	Weekend	Overall	Weekday^a^	Weekend^a^
Bedtime							0.17^b^	0.25^c^	0.02
Sleep onset time	0.38^c^	0.45^c^	0.07	0.85^c^	0.79^c^	0.94^c^	0.39^c^	0.49^c^	0.07
Sleep latency							0.04	0.07	0.02
Sleep end time	0.53^c^	0.64^c^	0.18^b^	0.83^c^	0.81^c^	0.70^c^	0.51^c^	0.59^c^	0.08
Sleep period	0.29^c^	0.27^c^	0.09	0.57^c^	0.43^c^	0.73^c^	0.35^c^	0.38^c^	0.01
Night wakings, no.	0.03	0.06	0.00	0.02	0.01	0.03	0.00	0.00	0.00

^a^Sleep patterns recorded on questionnaires over the entire week were also compared with weekday/weekend variables on sleep logs and actigraphy.

R-squares and *P*-values (^b^*P* < 0.01, ^c^*P* < 0.001) from Pearson’s correlation coefficient are presented without correction; a *P*-value less than 0.008 was considered to indicate statistical significance; Bonferroni correction was used to adjust for multiple comparisons for the 6 sleep variables, but not for other parameters.

**Table 2.** Variation in sleep patterns recorded in sleep logs, questionnaires, and by actigraphy

		Overall			Weekdays			Weekend		
				
		Sleep log	Questionnaire	Actigraphy	Sleep log	Questionnaire^a^	Actigraphy	Sleep log	Questionnaire^a^	Actigraphy
Bedtime	mean		21:09	21:39			21:32			22:01
	95% CI		21:01–21:18	21:28–21:49			21:21–21:42			21:45–22:16
	*P*		<0.001			<0.001			<0.001	

Sleep onset time	mean	21:31	21:32	21:47	21:25		21:40	21:45		22:08
	95% CI	21:22–21:40	21:23–21:40	21:36–21:58	21:16–21:35		21:29–21:51	21:31–22:00		21:53–22:24
	*P*^b^	<0.001	ns		0.004	ns		<0.001	0.001	
	*P*	<0.001	0.001		<0.001	ns		<0.001	<0.001	

Sleep latency	mean		0:22	0:08			0:09			0:08
	95% CI		0:18–0:27	0:07–0:09			0:07–0:10			0:06–0:09
	*P*		<0.001			<0.001			<0.001	

Sleep end time	mean	7:22	7:17	7:13	7:16		7:07	7:29		7:24
	95% CI	7:13–7:30	7:10–7:24	7:05–7:22	7:08–7:25		6:59–7:15	7:16–7:41		7:11–7:37
	*P*	<0.001	ns		<0.001	<0.001		ns	ns	

Sleep period	mean	9:49	9:45	9:26	9:51		9:26	9:38		9:15
	95% CI	9:40–9:56	9:36–9:54	9:18–9:34	9:42–10:00		9:17–9:35	9:25–9:51		9:02–9:29
	*P*	<0.001	<0.001		<0.001	<0.001		<0.001	<0.001	

Night wakings	mean	0.05	0.23	3.47	0.04		3.52	0.09		3.33
	95% CI	0.02–0.09	0.11–0.36	2.90–4.03	0.01–0.07		2.89–4.15	0.01–0.16		2.70–3.96
	*P*	<0.001	<0.001		<0.001	<0.001		<0.001	<0.001	

Times are presented as hh:mm.

^a^Sleep patterns on the questionnaire for the entire week were also compared with weekday/weekend variables on sleep logs and actigraphy. *P*-values from paired *t*-tests are presented without correction in comparisons with actigraphic data in the same column ^b^except for comparisons with actigraphic bed time.

A *P*-value less than 0.008 was considered to indicate statistical significance; Bonferroni correction was used to adjust for multiple comparisons for the 6 sleep variables, but not for other parameters.

### Comparison of the questionnaire and actigraphy

When actigraphy and the questionnaire were compared, bedtime, sleep onset time, sleep end time, and sleep period were all correlated for the whole study period and for weekdays (*P* = 0.004 for bedtime during the whole period; *P* < 0.001 for all other comparisons), but not for weekends. There was no significant correlation in sleep latency and number of night wakings between actigraphy and the questionnaire, regardless of the study period (Table 1). As compared with actigraphic observations, the questionnaire noted earlier bedtime, longer sleep latency and sleep period, and fewer night wakings, regardless of the study period (*P* < 0.001 for all comparisons; Table 2). For weekdays only, the sleep end time on the questionnaire was later than that yielded by actigraphy (*P* < 0.001, Table 2).

### Comparison of the sleep log and actigraphy

When actigraphy and sleep logs were compared, sleep onset time, sleep end time, and sleep period were all significantly correlated, regardless of the study period (*P* < 0.001 for all comparisons); there was no correlation with respect to the number of night wakings (Table 1). Sleep onset time recorded in sleep logs was earlier than both the bedtime and the sleep onset time recorded on actigraphy, regardless of the study period (*P* = 0.004 for the comparison between sleep onset time in the sleep log and bedtime on actigraphy during weekdays; *P* < 0.001 for all other comparisons; Table 2). In contrast, the sleep end time on actigraphy was earlier than that recorded in sleep logs for the whole period and for weekdays (*P* < 0.001 for both), but not for weekends (Table 1). As a result, the sleep period for actigraphy was shorter than that recorded in sleep logs, regardless of the study period (*P* < 0.001 for all comparisons; Table 2). The sleep log showed significantly fewer night wakings than did actigraphy (*P* < 0.0001, Table 2).

## DISCUSSION

The current study confirmed that there were significant linear correlations in sleep schedule variables recorded by actigraphy and in subjective sleep assessment tools in preschool children. However, in subjective sleep assessments, parents reported earlier sleep onset times, later sleep end times, and consequentially longer sleep periods than those recorded by actigraphy. Given that actigraphy typically overestimates sleep status, when compared with polysomnography,^23^ it is likely that parent-reported sleep periods contain significant wake time, because of the limited quality and continuity of parental observation. The extremely low detection rate for night wakings on parental reports was consistent with this hypothesis. Subjective sleep assessment tools based on parental reports are still in high demand for studies of preschool children; however, our current findings indicate that considerable care is required in interpreting such findings.

### Limitations of the study

Our study was based on a mixed population from 2 nurseries open to families with different child care needs. The majority of children in nursery B attended the nursery even on Saturday. Although nursery attendance on Saturday is popular in Japan, the weekend sleep patterns of such children might differ from those of children who have 2 weekend nights at home. The type of child care provided may further affect sleep patterns; however, our preliminary analysis suggested that the difference in nurseries did not affect the correlations between sleep variables obtained using the 3 assessment tools (data not shown). An extensive study investigating the influences of family lifestyle and other environmental factors in the same study population is currently underway. In addition, we used actigraphy as an alternative to the reference standard—polysomnography—to examine the characteristics of subjective sleep assessment tools. However, actigraphy relies solely on physical movement to indirectly assess sleep status, and hence is not equivalent to polysomnography. These limitations should be considered when comparing the present findings with those of other studies.

### Utility of actigraphy as an objective reference

Actigraphs have been accepted as convenient, minimally burdensome sleep assessment tools that provide objective information about sleep patterns. Actigraphy has now been validated for a wide range of subjects, including children with or without sleep disorders.^12^ Despite the increasing number of studies that have utilized actigraphy for the assessment of infants and school children, only a few have examined the sleep patterns of preschool children.^13^^–^^15^ Although we anticipated that preschool children might be too curious or impatient to tolerate the wristwatch devices, data were successfully collected for all participants in our study. Our results showed robust linear correlations in sleep schedule parameters recorded by actigraphy and subjective standard tools. The actigraphs used in our study are less expensive than polysomnography; however, in Japan, it still costs more than US$25 000 to introduce a system with licensed software and 15 watch devices. The high cost of actigraphy is likely to result in considerable continued demand for subjective sleep assessment tools.

### Characteristic features of parental sleep assessment

#### Comparison with actigraphy

Subjective sleep assessments based on parental reports had been used long before the establishment of polysomnographic evaluation. Agreement between sleep log and objective assessment tools in young children has already been demonstrated.^13^^–^^15^^,^^17^ However, only a few studies have addressed the nature of the correlations between different assessment tools. Our findings build on previous studies of relatively short duration (1–3 days) by Goodwin, Sekine, and their colleagues^24^^,^^25^: we observed significant linear relationships in sleep schedule variables between actigraphic data and data from parental reports. Our study also identified subtle differences between parental reports and actigraphic results: as compared with the actigraphic data, parents reported earlier sleep onset times, later sleep end times, and, consequently, longer sleep periods in the sleep logs and questionnaires. Although actigraphic sleep assessment is objective, actigraphy only indirectly estimates sleep patterns, and there are not enough data to support the notion that actigraphy is more reliable than subjective assessment tools in assessing sleep status. However, because actigraphy typically identifies longer sleep periods than does polysomnography,^23^ it is very likely that parental observation considerably overestimates sleep status.

#### Potential bias in parental reports

Sekine and colleagues speculated that parents may misidentify a part of sleep latency as sleep status, thus resulting in parental reports that estimate a longer sleep period than that noted in actigraphic data.^25^ In our study, sleep onset in sleep logs was even earlier than actigraphic bedtime. This finding suggests that the period defined as sleep latency on actigraphy is generally thought to be sleep itself on parental observation, presumably because of the limited sensitivity of parental observation. This trend was clearly demonstrated by the fact that parents reported fewer than 10% of the night wakings detected on actigraphy.

In addition to the limitations associated with parental observation, psychological biases may also influence parental reports, especially when parents rely on their memory to record abbreviated sleep patterns rather than punctually recording them in the daily log. In our current study, the sleep period reported on the questionnaire generally well correlated with objectively assessed values. However, in the questionnaire, parents tended to report relatively longer (shorter) sleep periods when their children’s sleep periods were extremely short (long) (Online supplementary 3–4). Although it would appear that punctual daily data-logging makes a sleep log less vulnerable to subjective modification of data than would a questionnaire, psychosocial pressures might also affect the information entered into sleep logs. When interpreting parental sleep assessments, it is therefore essential to carefully account for such characteristics of the assessment tool.

### Conclusions

Sleep schedule variables on parental reports significantly differed from actigraphic observations. However, parental reports and actigraphy showed strong linear correlations, presumably because parental reports consistently overestimated sleep status. This information about the characteristics and potential biases of such assessments should be utilized to enable the precise interpretation and comparison of findings.

Our results suggest different possible uses for subjective sleep assessments and actigraphy. Handy actigraphs were tolerated even by young preschool children, and yielded objective sleep variables. However, actigraphs are expensive, and may require further validation by polysomnography in infants and young preschool children. Sleep logs produce reliable sleep schedule data without the use of expensive devices, and hence would be preferable, especially for large surveillance studies. Parental assessment, however, should not be preferred over actigraphy for studies that address the quality of sleep, because parental reports appeared to be insensitive to night wakings. In situations where research resources are limited, the use of brief questionnaires might still be effective, depending on the purpose and size of the study. Our study used a brief sleep questionnaire in which parents were asked to describe the habitual sleep patterns of children, without differentiating weekdays and weekends. Although such an abbreviated questionnaire is commonly used for infants, the sleep schedule variables given by the questionnaire agreed with actigraphic sleep schedule variables on weekdays but not weekends, suggesting that parents usually consider weekday sleep as habitual sleep. Future studies of preschool children should attempt to obtain information specific to weekdays and weekends.

## ONLINE ONLY MATERIALS

eMaterial.
