# Supplementary material for: Utility of Subjective Sleep Assessment Tools for Healthy Preschool Children: A Comparative Study Between Sleep Logs, Questionnaires, and Actigraphy
Source: J Epidemiol. 2010 Mar 5;20(2):143–9. doi: 10.2188/jea.JE20090054 (PMC3900813; doi:10.2188/jea.JE20090054)
Supplement: eMaterial. [file je-20-143-s001.pdf]

Online supplementary 1:
Representative sleep record from sleep log and actigraphy

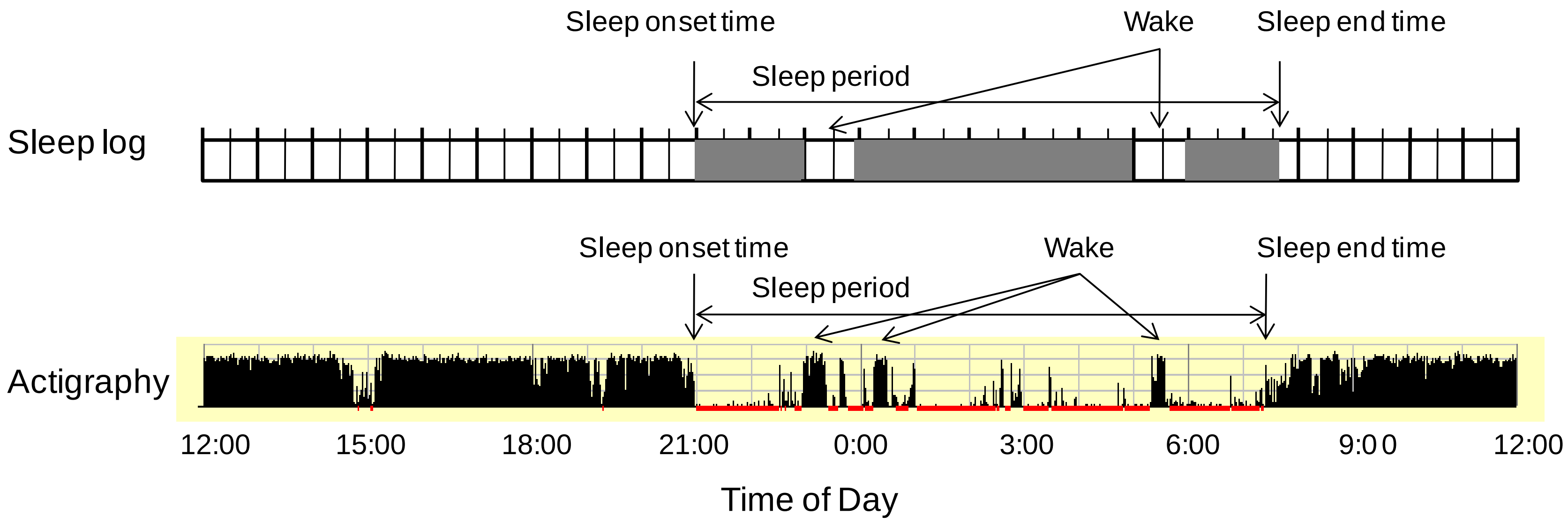

A 24-hour record from a representative subject demonstrates a sleep pattern on a sleep log and on actigraphy. The sleep log has been reproduced from the raw reporting sheet: open rectangles represent wakefulness whereas grey rectangles represent sleep. Dark areas on the actigraphic raw data are associated with daytime activity and nocturnal wakefulness.

Online supplementary 2:
Full detail of brief questionnaire

Please mark only one most appropriate answer referring to your child's habitual sleep for the last four weeks.

Name of responder: ________________ Date: ___________
Role of Responder: ☐ Father ☐ Mother

A. Sleep arrangement:
   - ☐ Child's own bed (or equivalent sleeping surface) in separate room
   - ☐ Child's own bed (or equivalent sleeping surface) in parents' room
   - ☐ In parents' bed (or equivalent sleeping surface)
   - ☐ Other (please specify): ______________________________________

B. In what position does your child sleep most of the time?
   ☐ On his/her belly ☐ On his/her side ☐ On his/her back

C. How much time does your child spend in sleep during the night?
   ( ) hr ( ) min

D. What time does your child get into bed? ( ) hr ( ) min

E. What time does your child fall asleep actually? ( ) hr ( ) min

F. What time does your child wake up in the morning? ( ) hr ( ) min

G. Is the bath time of your child fixed or flexible? ☐ Yes ( ) hr ( ) min
   ☐ No

H. How many night wakings (longer than 5 minutes) are usually noticed per night with your child?
   ( )

I. How much time during the night does your child spend in wakefulness?
   ( ) hr ( ) min

J. Is your child's sleep pattern stable? ☐ Yes ☐ No

K. (Only for parents who answered No to Question J)
   Who is the most responsible for child's unstable sleep patterns? :
   ☐ Child him/herself ☐ Parents ☐ Unknown

L. Do you consider your child's sleep as a problem?
   ☐ A very serious problem ☐ A small problem ☐ Not a problem at all

The brief infant sleep questionnaire developed by Sadeh (Sadeh, 2004) was modified and translated into Japanese (only the draft before translation is shown) to obtain the abbreviated sleep patterns of preschool children over a four-week period.

Online supplementary 3:
Correlation between reported and calculated sleep periods on questionnaire

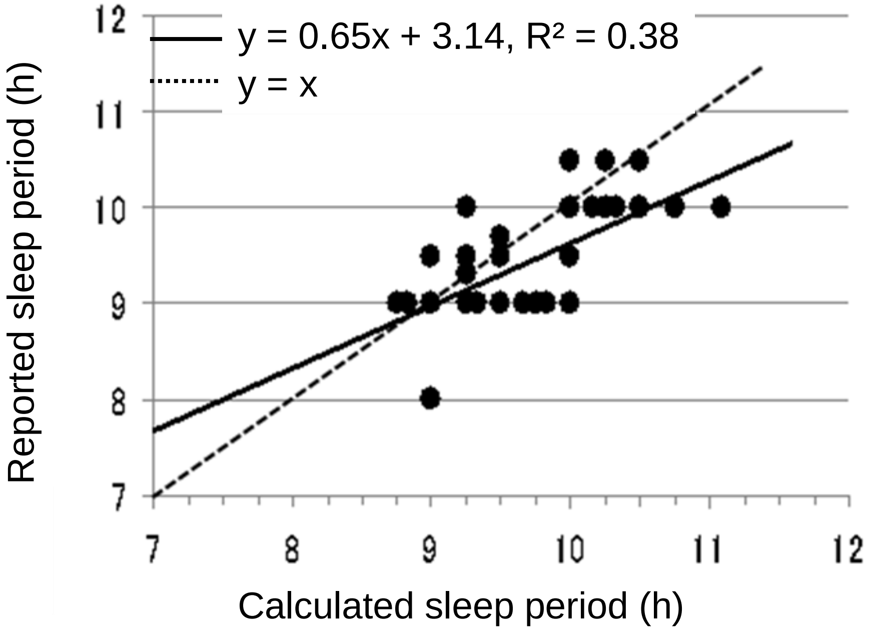

To investigate potential bias on the questionnaire, the “calculated sleep period” was given from “sleep onset time” and “sleep end time” in the questionnaire data; this was compared with the reported “sleep period”. There was a significant linear correlation between the reported- and calculated- sleep periods ($p < 0.001$). However parents tended to report longer sleep periods than the calculated values when their children had below-average sleep periods, resulting in a positive constant of 3.1 hours for the regression equation.

Solid line: regression line for the scatter between the reported and calculated sleep periods. Broken line: reference line for the equation of ***y* = *x***, which is expected when two sleep periods are equivalent and identical.

Online supplementary 4: Estimation error in parental assessment and actigraphic sleep period

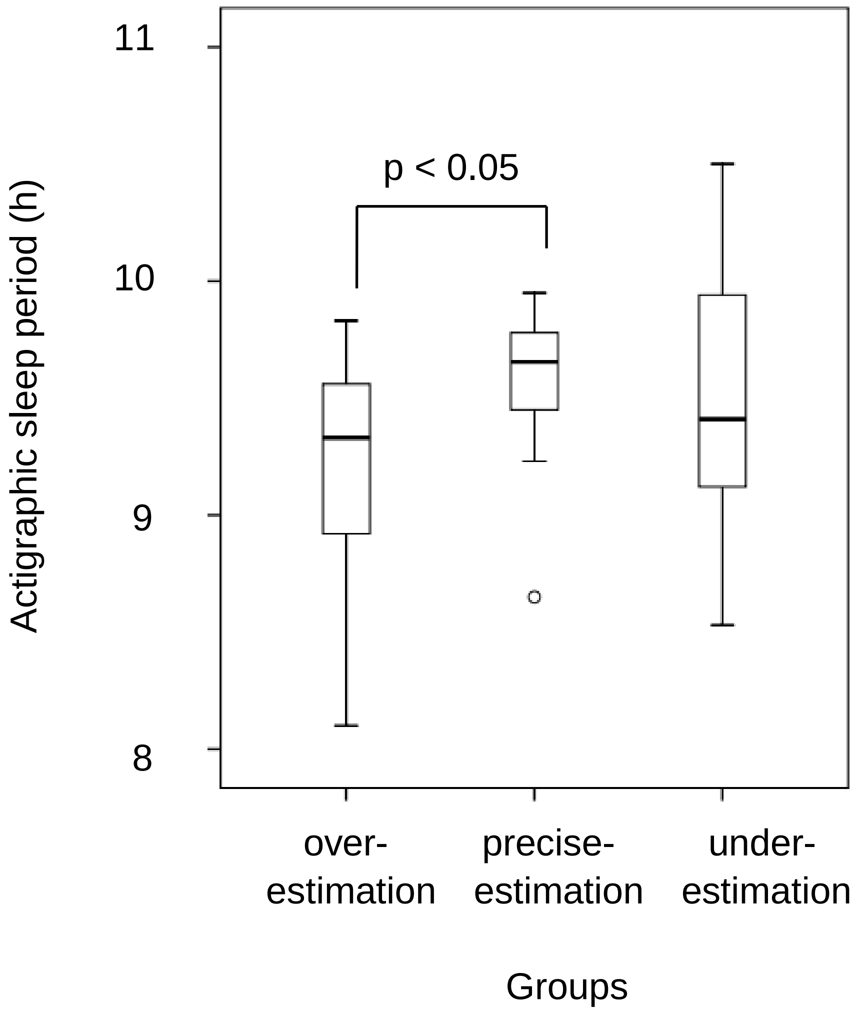

Within the three groups whose parents reported sleep periods which were longer than (over-estimation group, 10 minutes < difference, n = 9), similar to (precise-estimation group, -10 minutes < difference ≤ 10 minutes, n = 12) and shorter than (under-estimation group, difference ≤ -10 minutes, n = 26) the calculated sleep periods (see online supplementary 3), the actigraphic sleep period in the over-estimation group was shorter than that in the precise-estimation group ($p < 0.05$).

Symbols: box - first and third quartiles, bold line - median, perpendicular line - range without outliers, open circle - extreme outlier less than 1.5 times the interquartile range from the first quartile.
